# Do non-local hospitalized patients cost more? A comparative study based on the implementation of the DRG payment

**DOI:** 10.3389/fpubh.2026.1841847

**Published:** 2026-05-29

**Authors:** Wenbo Du, Xuxian Ren, Yaxin Liu, Hui Yu, Yuying Luo, Xin Yao, Jin Wen

**Affiliations:** 1 Chongqing Medical University-University of Leicester Joint Institute, Chongqing Medical University, Chongqing, China; 2 Institute of Hospital Management, West China Hospital of Sichuan University, Chengdu, China; 3 Sichuan Health Information Center, Chengdu, China

**Keywords:** cost-shifting, DRG payment, healthcare expenditure, non-local patients, total hip arthroplasty (THA)

## Abstract

**Aims:**

This study investigates whether non-local patients—those hospitalized outside their registered insurance region—incur higher medical expenditures than local patients under China’s Diagnosis-Related Groups (DRG) payment reform. Total hip arthroplasty (THA) was used as a standardized clinical model to evaluate cost disparities and potential cost-shifting behaviors post-reform.

**Methods:**

We analyzed 55,532 THA inpatient records from Sichuan Province (2015–2023), classifying patients as local or non-local. Descriptive statistics and univariate tests were conducted using R and SPSS. A multi-period difference-in-differences (DID) model was employed to estimate the reform’s impact, adjusting for individual, institutional, and temporal variables.

**Results:**

Non-local patients consistently incurred higher hospitalization costs, despite being younger and having fewer comorbidities. Prior to DRG implementation, the average cost gap was CNY 2,730, mainly from treatment and examination fees. Post-DRG, the gap widened to CNY 2,869 (*p* < 0.01), with significant increases across all categories—especially consumables and treatment. DID analysis showed significant cost reductions for local patients, while treatment costs for non-local patients rose (*β* = 0.11, *p* < 0.01), suggestive of patterns consistent with potential cost-shifting behavior under differential payment models.

**Conclusion:**

DRG payment reform effectively reduced costs for local patients but was linked to selective cost increases for non-local patients, particularly in treatment-related spending. These findings suggest that mixed reimbursement models may incentivize differential billing. Ongoing monitoring of expenditure structures is crucial to ensure equitable policy outcomes.

## Background

Diagnosis-Related Groups (DRG) payment systems have been widely adopted globally, especially in high-income countries, as an important mechanism to enhance hospital efficiency, optimize resource allocation, and control healthcare expenditures (1). With growing health spending and health insurance payment system reforms, controlling medical costs has become a critical issue within public health and healthcare policies (2, 3). Currently, China is implementing the DRG-based reimbursement system gradually for local insured patients, aiming at optimizing healthcare spending and improving hospital productivity (4, 5).

With the implementation of the DRG payment policy, the question of whether health care costs and health care services for groups outside of Medicare coverage are also affected has been widely explored (6, 7). In particular, China is currently experiencing an uneven development of healthcare resources. Due to the uneven distribution of healthcare resources, non-local patients refer to individuals receiving medical services outside their registered insurance locations, mainly characterized by those who travel across cities for medical care. However, non-local insured patients typically follow a Fee-For-Service (FFS) payment model, which raises concerns about potential disparities in costs and treatment effects (8).

While the DRG system has been proven effective in reducing the length of hospital stays (LOS) and enhancing efficiency, some studies also explored cost-shifting for FFS patients after DRG implementation (9, 10). Impacts on non-local patient costs and received healthcare remain inadequately studied (11).

In China, basic medical insurance is pooled and administered at the prefecture-level city (12), meaning that a patient’s reimbursement eligibility is determined by the city in which they are enrolled rather than the city in which they receive care. Patients hospitalized within their enrolment city are reimbursed under the local insurance scheme; those who seek care across city boundaries are instead reimbursed on a FFS basis, as cross-city DRG payment arrangements were not yet operational during the study period. The latter was referred to as non-local patients. In Sichuan Province, DRG prospective payment was introduced on a phased, city-specific basis from 2019 onwards, reaching province-wide coverage by 2021. Consequently, throughout the study period (2015–2023), local patients were progressively incorporated into DRG bundled payment with fixed per-case budget constraints, while non-local patients remained under FFS reimbursement without equivalent per-episode caps. This structural payment asymmetry between the two patient groups coexisted within the same provider institutions and formed the institutional mechanism central to the present study.

Total hip arthroplasty (THA) is a relatively mature surgical method for the treatment of hip joint diseases in orthopedics. With the increasing aging of the population and the continuous progress of hip replacement technology, the number of THA cases is also increasing continuously (13). Some studies based on national data have shown that hip replacement bundled payment led by doctors is more efficient (14). This paper takes THA as the research object to explore the changes in the costs of local and non-local patients after the implementation of the DRG payment and whether there is any cost-shifting behavior by physicians. By exploring the changes in medical costs and cost structure among local patients after the implementation of DRG and possible cost-shifting among non-local patients, can validate the effectiveness of the DRG payment and provide suggestions for further adjustments to the DRG payment to promote the balance of quality, efficiency, and fairness.

## Methods

### Data source and processing

Data analyzed in this study were sourced from medical records of patients undergoing total hip arthroplasty (THA) in Sichuan Province from 2015 to 2023. Patients who underwent THA surgery classified under ICD-9 procedure code 81.51 were included.

Patients were classified as local if hospitalized within their insurance enrolment city, and non-local if hospitalized outside it (10, 15, 16). Thus, those who crossed city boundaries to seek inpatient care within Sichuan Province were categorized as non-local. This distinction reflects the administrative structure of China’s basic medical insurance system, under which reimbursement eligibility and payment modality are determined at the prefecture-level city. As insurance enrolment city is not directly recorded in hospital discharge records, we used the city corresponding to the patient’s registered domicile (hukou location) as an operational proxy, consistent with established practice in cross-regional healthcare research in China.

The exclusion criteria were as follows:

- Missing data: Medical records lacking critical data (e.g., identification number, residence information, or medical payment method).
- Outlier removal: Records with continuous outcome measures (total hospitalization costs, length of stay) outside the 1st to 99th percentile range.
- Logical errors: Data with internal inconsistencies, such as discrepancies between patient age and date of birth, mismatches between diseases and patient age, or abnormal admission-discharge counts.

A total of 65,126 patient records were initially enrolled in this study. Following exclusion criteria, 31 duplicates were excluded, and 8,030 records with missing values in clinically critical variables were removed. Additionally, 690 records exhibiting logical inconsistencies were discarded. To address extreme values, we applied 1% winsorization to 843 outlier records for key expenditure variables. The final analytical cohort comprised 55,532 cases. All expenditure metrics were inflation-adjusted using the Consumer Price Index (CPI) for Sichuan Province (source: Sichuan Provincial Bureau of Statistics), with 2015 as the base year. Log-transformation was applied to approximate normality, thereby ensuring the validity of parametric statistical analyses. All data cleaning procedures were performed using R software.

### Statistical analysis

The primary outcome variables were total hospitalisation costs and four cost subcategories: drug costs, examination costs, treatment costs, and consumables costs. Consumables costs refer to expenses for surgical implants, disposable medical supplies, and other one-time-use materials used during the procedure, as recorded in the hospital discharge record.

For data description, normality of variables was assessed via the Shapiro–Wilk test (*α* = 0.05) using both R and SPSS. Continuous variables conforming to normal distribution were summarized as mean ± standard deviation, while non-normally distributed variables were expressed as median with interquartile range (IQR). Univariate analyses were conducted in SPSS to explore preliminary associations between costs and DRG payment. Independent two-sample t-tests were applied for normally distributed variables, with Mann–Whitney U tests employed as non-parametric alternatives where normality assumptions were violated.

For empirical analysis section, considering the phased, city-specific introduction of the DRG payment, this study employed a multi-period difference-in-differences (DID) model (17). Since the DRG payment was not withdrawn or reversed once implemented, the multi-period DID method was selected to better capture the dynamic implementation effects. The multi-period DID model constructs multiple separate treatment and control datasets for each policy pilot period, stacking them into one integrated dataset, upon which standard DID regression analyses were performed. The model controlled for space (city) and time (year) fixed effects, as well as individual characteristics such as gender, age, Charlson Comorbidity Index (CCI), payment methods, and hospital types. Insurance type (Resident versus Employee Medical Insurance) was included as a covariate in all models; as payment modality was time-invariant for non-local patients throughout the study period, a locality-by-payment-mode interaction term was not estimable and was therefore excluded from the model specification.

The multi-period DID model is specified as follows:

$$Y_{\mathrm{ict}}=\sigma +\alpha_{c}+\lambda_{t}+\beta_{0}(D_{\mathrm{ct}}\times T_{\mathrm{ct}})+\beta_{1}X_{\mathrm{ict}}+\varepsilon_{\mathrm{ict}}$$

In the above equation, the subscripts i,c,t represent patient, city (region of treatment), and year, respectively. The terms $\alpha_{c}\;$ and $\lambda_{t}\;$ denote city fixed effects and year fixed effects, respectively, controlling for city-level characteristics that are either time-invariant or vary only over time. The key explanatory variable in this multi-period setting is $D_{\mathrm{ct}}\times T_{\mathrm{ct}}$ , indicating whether city *c* implemented the DRG pilot payment in year *i*. Individual-level characteristics $X_{\mathrm{ict}}$ , such as gender, age, Charlson Comorbidity Index (CCI), and medical payment methods, were included as control variables. Finally, $\varepsilon_{\mathrm{ict}}$ represents the random error term. The staggered rollout of DRG across prefecture-level cities provides the identifying variation for the multi-period DID design, with different cities adopting the reform at different time points between 2019 and 2021. City fixed effects absorb time-invariant differences in healthcare infrastructure and baseline cost levels across cities, while year fixed effects control for province-wide temporal trends common to all cities. This design ensures that the estimated policy effects reflect within-city changes in costs following DRG implementation, rather than pre-existing differences between cities or concurrent secular trends.

## Results

### Basic information

**Figure 1:**
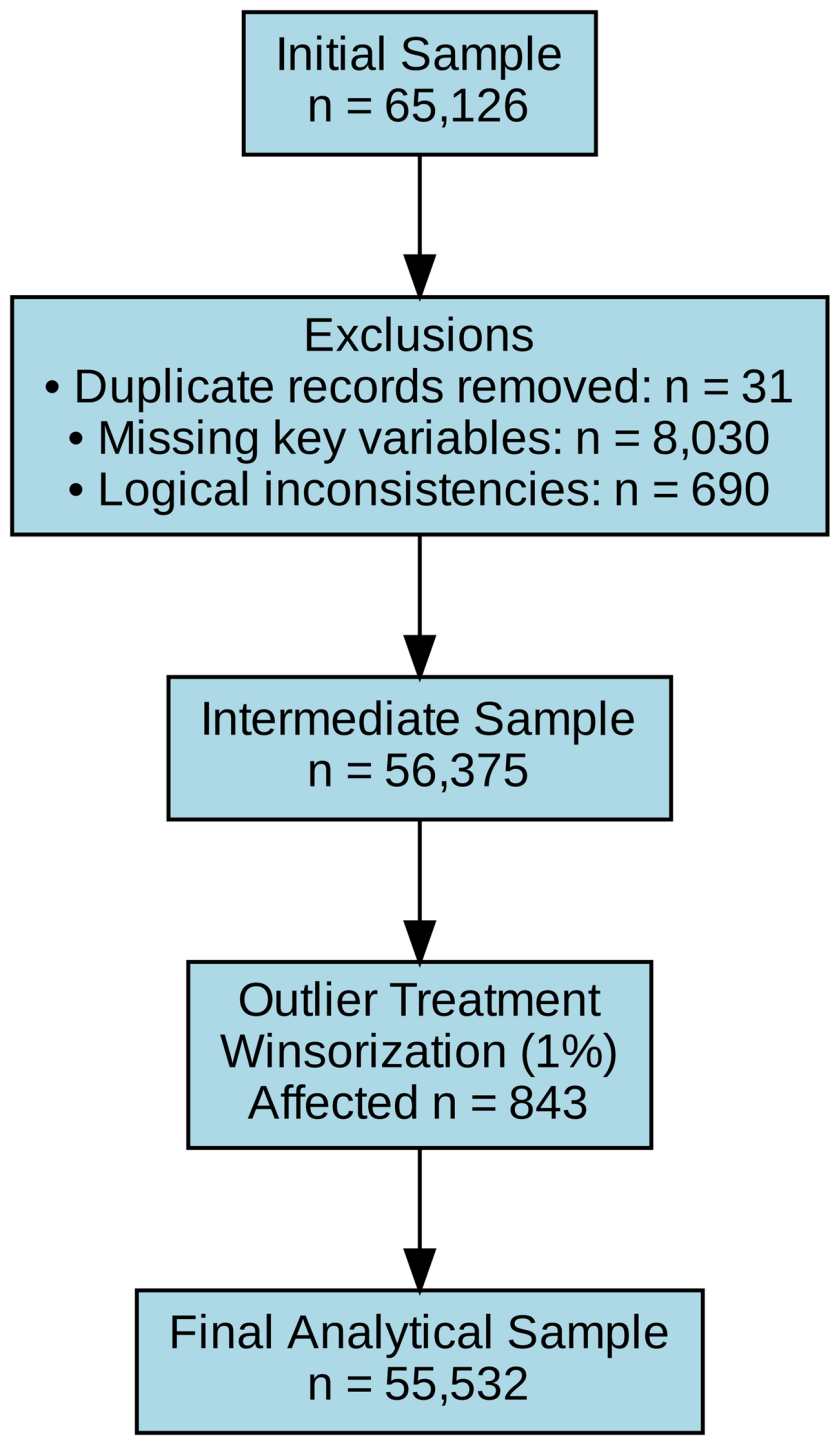
Flowchart of patient selection and sample processing.

The complete patient selection process is illustrated in Figure 1. Following sequential application of the exclusion criteria, 1% winsorization was applied to key expenditure variables to address extreme values. The final analytical cohort comprised 55,532 cases.

Table 1 displays patient demographic and clinical characteristics. Gender differences between local and non-local patients were insignificant (*p* = 0.06), while significant differences existed in age distribution, payment methods, length of stay (LOS), hospital types, CCI, and clinical pathway management (all *p* < 0.01). Non-local patients were significantly younger, preferred specialty hospitals, and generally presented fewer comorbidities compared to local patients.

**Table 1:** Baseline characteristics for local and non-local patients (*n*/%).

Variable	Local patients *n* (%)	Non-local patients *n* (%)	*p*-value
Gender			
Male	25,181 (51.39)	3,278 (50.18)	0.06
Female	23,819 (48.61)	3,254 (49.82)	
Age (years)			
≤50	7,188 (14.49)	1859 (28.39)	<0.01
50–	19,687 (39.69)	2,743 (41.89)	
65–	20,396 (41.12)	1747 (26.67)	
≥80	2,327 (4.69)	199 (3.04)	
Length of Stay (days)			
≤8	4,706 (9.49)	2059 (31.44)	<0.01
8–	21,644 (43.64)	2,864 (43.74)	
16–	18,938 (38.18)	1,287 (19.65)	
≥24	4,310 (8.69)	338 (5.16)	
Medical payment method			
Resident Medical Insurance	36,148 (73.77)	4,282 (65.55)	
Employee Medical Insurance	12,852 (26.23)	2,250 (34.45)	<0.01
Hospital type			
General Hospital	30,837 (62.93)	4,723 (49.82)	<0.01
Specialty Hospital	5,956 (12.16)	684 (72.31)	
Traditional medicine hospital	12,202 (24.90)	1,125 (17.22)	
CCI index			
0	31,932 (65.17)	4,659 (71.33)	<0.01
1	11,937 (24.36)	1,352 (20.70)	
2	3,703 (7.56)	372 (5.70)	
≥3	1,428 (2.91)	149 (2.28)	
Clinical pathway management	19,725 (39.77)	2058 (31.5)	<0.01

The distribution of Medical Payment Method (Resident Medical Insurance vs. Employee Medical Insurance) across local and non-local patient groups reflects differences in insurance-type composition between the two cohorts (*p* < 0.01). Insurance type was included as a covariate in all regression models to control for its potential confounding effect on the locality-cost relationship.

**Figure 2:**
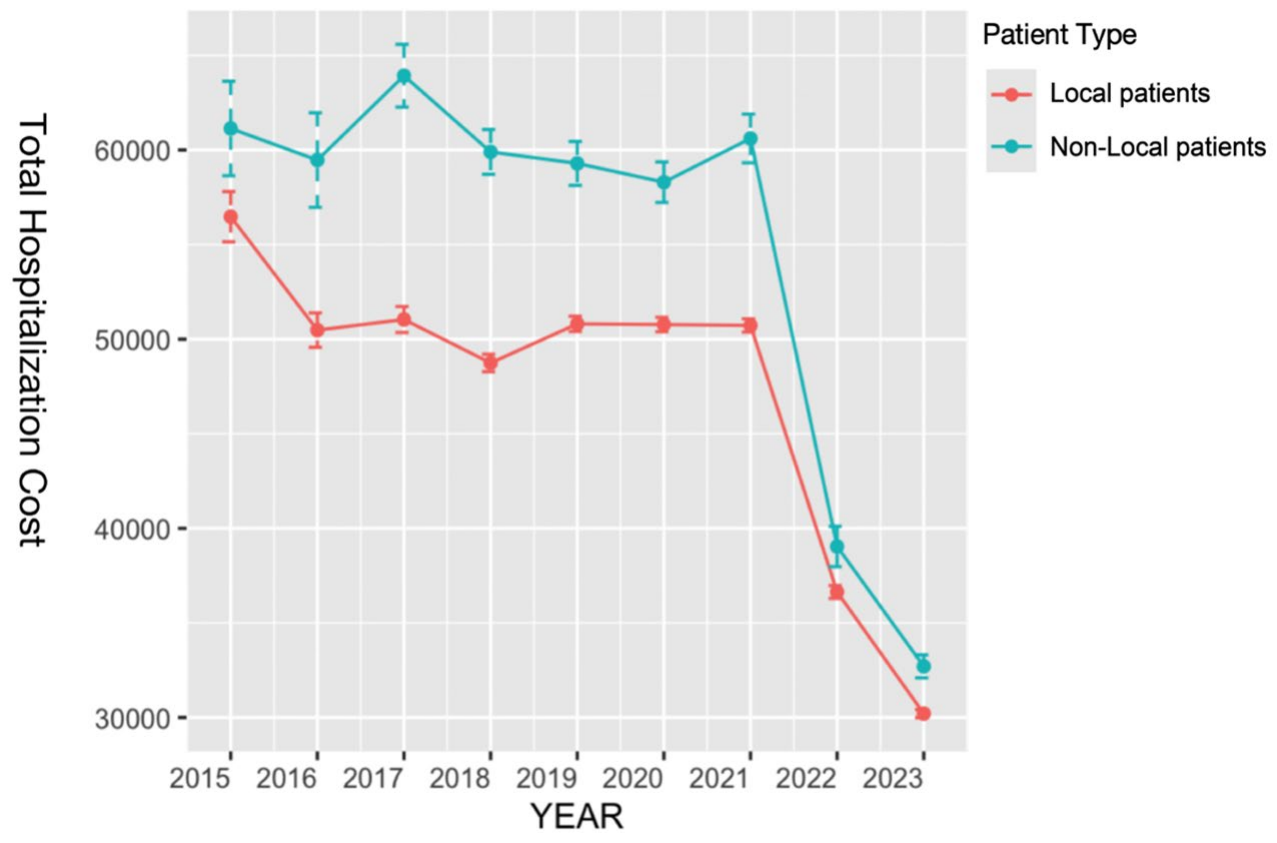
Trends in total hospitalization costs for local and non-local patients undergoing THA from 2015 to 2023.

Figure 2 illustrates the longitudinal trends in total hospitalization costs for local and non-local patients undergoing THA between 2015 and 2023. Over the nine-year period, non-local patients consistently incurred higher total hospitalization costs compared to local patients. This disparity persists despite baseline characteristics (Table 1) indicating that non-local patients were younger, had fewer comorbidities, and shorter average hospital stays—factors typically associated with reduced expenditures. Such a divergence may suggest systemic cost-shifting literature associates with mixed payment environments, FFS-reimbursed patients may bear a disproportionate share of costs relative to DRG-capped local patients. A notable inflection point occurred in 2021, with a sharp decline in costs for both cohorts. This temporal alignment coincides with the province-wide implementation of the DRG policy in Sichuan Province.

### Changes in medical costs pre- and post-DRG

For Pre-DRG period, non-local patients incurred significantly higher total hospitalization costs compared to local patients (50,525 CNY vs. 47,795 CNY, *p* < 0.01), driven primarily by elevated examination costs (4,388 CNY vs. 3,949 CNY, *p* < 0.01) and treatment costs (8,863 CNY vs. 6,379 CNY, *p* < 0.01). Notably, consumables costs for non-local patients were marginally lower (*Δ* = −218.98, *p* > 0.10), while medication costs showed no statistically significant disparity.

After the DRG payment policy was implemented, the cost gap widened substantially, with non-local patients exhibiting CNY 2,868.82 (*p* < 0.01) higher total hospitalization costs. All subcategories demonstrated significant disparities: medication (*Δ* = CNY 371.31, *p* < 0.01), examination (*Δ* = CNY 136.81, *p* < 0.01), treatment (*Δ* = CNY1 306.01, *p* < 0.01), and consumables (*Δ* = CNY 1,065.54, *p* < 0.01). Strikingly, the post-policy consumables costs reversed their pre-policy trend, becoming significantly higher for non-local patients, which may reflect differential cost allocation patterns consistent with potential cost-shifting behaviors (see Table 2).

**Table 2:** Changes in costs for local and non-local patients before and after the implementation of the DRG policy.

Cost category	Before DRG policy (FFS)			After DRG policy (pay for DRG)		
	Local patients (*n* = 49,000)	Non-local patients (*n* = 6,532)	mean-diff	Local patients (*n* = 16,932)	Non-local patients (*n* = 970)	mean-diff
Total Hospitalization Costs (CNY)	47795.96 (17393.39)	50525.94 (19789.97)	2729.98***	34574.27 (14726.55)	37443.09 (18801.51)	2868.82***
Drug Costs (CNY)	4106.92 (2641.60)	4251.27 (3195.06)	144.35	3217.41 (2156.93)	3588.72 (2297.85)	371.31***
Examination Costs (CNY)	3949.87 (2254.43)	4388.30 (2138.71)	438.43***	3649.18 (1795.44)	3785.99 (1788.22)	136.81***
Treatment Costs (CNY)	6379.95 (3553.99)	8863.54 (3323.11)	2483.59***	5553.67 (2644.27)	6859.68 (2920.65)	1306.01***
Consumables Costs (CNY)	26606.76 (17152.61)	26387.78 (19484.49)	−218.98	18635.27 (13656.29)	19700.81 (16247.48)	1065.54***

***p < 0.01, *p < 0.05, *p < 0.10.

### Multi-period DID regression analysis

The multi-period DID analysis demonstrates distinct policy-driven behavioral shifts across cost categories (Table 3). For local patients, the DRG policy significantly reduced total hospitalization costs (*β* = −0.01, *p* = 0.03), primarily through declines in medication (*β* = −0.04, *p* = 0.01), examination (*β* = −0.07, *p* < 0.01), and treatment expenditures (*β* = −0.03, *p* < 0.01). However, consumables costs exhibited a paradoxical increase (*β* = 0.10, *p* = 0.03), likely reflecting hospitals’ strategic substitution toward higher-cost materials to offset DRG-imposed revenue constraints.

**Table 3:** Multi-period difference-in-differences regression results of DRG policy on hospitalization costs for local and non-local patients.

Model statistic	Total hospitalization costs		Drug costs		Examination costs		Consumables costs		Treatment costs	
	Local patients	Non-local patients	Local patients	Non-local patients	Local patients	Non-local patients	Local patients	Non-local patients	Local patients	Non-local patients
Coefficient (β)	−0.01**	0.007	−0.04**	0.05	−0.07***	−0.04	0.10**	0.009	−0.03***	0.11***
*t* value	−2.22	0.37	−2.48	1.26	−3.11	−0.87	2.25	0.07	−2.61	3.88
*p*-value	0.03	0.71	0.01	0.21	<0.01	0.39	0.03	0.94	<0.01	<0.01
*N*	49,000	6,532	49,000	6,532	49,000	6,532	49,000	6,532	49,000	6,532
*R*-squared	0.55	0.67	0.13	0.26	0.10	0.21	0.09	0.20	0.17	0.25

***p < 0.01, *p < 0.05, *p < 0.10.

Patient age, gender, CCI, length of hospital stay, clinical pathway management, payment method, and hospital type were controlled.

Among non-local patients, total costs and most subcategories showed no statistically significant post-policy changes. Notably, treatment costs surged disproportionately (*β* = 0.11, *p* < 0.01), suggesting expenditure patterns consistent with potential cost-shifting under differential payment incentives.

## Discussion

This study systematically evaluated the impact of Diagnosis-Related Groups (DRG) payment policies on medical expenditures among local and non-local patients undergoing total hip arthroplasty (THA), with particular emphasis on the potential phenomenon of cost-shifting.

This study revealed that the DRG payment policy effectively decreased overall hospitalization expenditures for local patients, particularly evident in pharmaceuticals, diagnostic tests, and treatment costs, which is consistent with international evidence supporting DRG’s efficacy in improving cost control (18, 19). However, the significant increase in consumables costs suggests preferential selection of higher-cost surgical implants, consistent with international evidence linking DRG adoption to increased use of premium-priced joint replacement components despite clinical equivalence (20).

Total expenditures for non-local patients did not significantly decline following DRG implementation, and treatment expenses increased significantly, a pattern consistent with potential cost-shifting behavior under differential payment incentives (10, 21, 22). One potential explanation relates to the structural difference in payment design: unlike DRG payment, FFS reimbursement imposes no per-episode budget ceiling, which may reduce provider incentive to contain costs for non-local patients irrespective of reform (2).

Non-local patients’ restricted access to alternative institutions and limited knowledge of local hospital pricing structures may represent contributing factors (23, 24); though differences in service intensity and provider practice patterns cannot be excluded with the available observational data. Confirming the underlying mechanism would require complementary data sources, such as provider billing records or procedure-level audit data, which are beyond the scope of the current administrative dataset.

Overall, the analysis indicates that the coexistence of DRG and FFS payment within the same provider institutions may generate different cost incentives across patient groups (25), with consequences that warrant policy attention. Notably, despite being younger and having fewer comorbidities, non-local patients still incurred higher hospitalization costs following DRG implementation increase was seen in treatment costs for non-local patients. The divergent expenditure trajectories observed between the two groups are consistent with patterns that the cost-shifting literature associates with mixed payment environments, though the observational design of this study precludes direct attribution of these changes to deliberate provider behavior.

There may be some possible limitations in this study. The study lacked longitudinal follow-up of patient health outcomes, potentially overlooking long-term implications of DRG implementation. And, the observational design and reliance on administrative discharge records preclude causal attribution of expenditure patterns to deliberate provider behavior or capture of qualitative provider decision-making; billing records or procedure-level audit data would be required to confirm the underlying mechanism. Third, local versus non-local classification relied on registered domicile (hukou) as a proxy for insurance enrolment city. Misclassification risk is mitigated by two considerations: the predominantly older, elective THA population has substantially lower hukou–insurance misalignment than younger working-age groups and, more critically, the uniform FFS reimbursement applied to all non-local patients regardless of home city preserves the payment-modality contrast central to the analysis. Fourth, hospitals actively conducting THA clinical trials may follow standardized protocols or use specific implants that affect cost structure differently from routine care; however, the large analytical sample spanning over 9 years and the inclusion of hospital-type fixed effects in all models mitigate the risk of systematic confounding from this source. Additionally, interaction terms between locality and insurance type were excluded because non-local patients’ FFS status was uniform and time-invariant, precluding identification within the DID framework; future studies capturing payment transitions may examine heterogeneous effects through subgroup designs.

Despite these limitations, the findings provide valuable insights for DRG payment implementation in other regions. Future research should explore targeted policy adjustments and institutional interventions to address expenditure disparities between local and non-local patients. Specifically, strategies such as refining insurance reimbursement standards, increasing transparency in inter-regional medical billing and strengthening regulation of medical practices are critical (26).

## Data Availability

The data analyzed in this study is subject to the following licenses/restrictions: the datasets analyzed during the current study are not publicly available due to data protection regulations, but are available from the corresponding author on reasonable request. Requests to access these datasets should be directed to huaxiwenjin@163.com.
